# The Anti-SARS-CoV-2 IgG1 and IgG3 Antibody Isotypes with Limited Neutralizing Capacity against Omicron Elicited in a Latin Population a Switch toward IgG4 after Multiple Doses with the mRNA Pfizer–BioNTech Vaccine

**DOI:** 10.3390/v16020187

**Published:** 2024-01-26

**Authors:** Ana M. Espino, Albersy Armina-Rodriguez, Laura Alvarez, Carlimar Ocasio-Malavé, Riseilly Ramos-Nieves, Esteban I. Rodriguez Martinó, Paola López-Marte, Esther A. Torres, Carlos A. Sariol

**Affiliations:** 1 Department of Microbiology and Medical Zoology, University of Puerto Rico-Medical Sciences Campus, San Juan, PR 00936, USA; albersy.armina@upr.edu (A.A.-R.); laura.alvarez@upr.edu (L.A.); carlimar.ocasio@upr.edu (C.O.-M.); riseilly.ramos@upr.edu (R.R.-N.); 2 Gastroenterology Research Unit, School of Medicine, University of Puerto Rico, San Juan, PR 00925, USA; esteban.rodriguez3@upr.edu (E.I.R.M.); paola.lopez.marte@gmail.com (P.L.-M.); estheratorresmd@gmail.com (E.A.T.); 3 Department of Medicine, University of Puerto Rico-Medical Sciences Campus, San Juan, PR 00936, USA; 4 Unit of Comparative Medicine, University of Puerto Rico-Medical Sciences Campus, San Juan, PR 00936, USA

**Keywords:** COVID-19, IgG4, class switch, ELISA, neutralizing antibody

## Abstract

The aim of this study was to analyze the profiles of IgG subclasses in COVID-19 convalescent Puerto Rican subjects and compare these profiles with those of non-infected immunocompetent or immunocompromised subjects that received two or more doses of an mRNA vaccine. The most notable findings from this study are as follows: (1) Convalescent subjects that were not hospitalized developed high and long-lasting antibody responses. (2) Both IgG1 and IgG3 subclasses were more prevalent in the SARS-CoV-2-infected population, whereas IgG1 was more prevalent after vaccination. (3) Individuals that were infected and then later received two doses of an mRNA vaccine exhibited a more robust neutralizing capacity against Omicron than those that were never infected and received two doses of an mRNA vaccine. (4) A class switch toward the “anti-inflammatory” antibody isotype IgG4 was induced a few weeks after the third dose, which peaked abruptly and remained at high levels for a long period. Moreover, the high levels of IgG4 were concurrent with high neutralizing percentages against various VOCs including Omicron. (5) Subjects with IBD also produced IgG4 antibodies after the third dose, although these antibody levels had a limited effect on the neutralizing capacity. Knowing that the mRNA vaccines do not prevent infections, the Omicron subvariants have been shown to be less pathogenic, and IgG4 levels have been associated with immunotolerance and numerous negative effects, the recommendations for the successive administration of booster vaccinations to people should be revised.

## 1. Introduction

The severe acute respiratory syndrome-related coronavirus 2 (SARS-CoV-2) pandemic and the resulting unprecedented outbreak of coronavirus disease 2019 (COVID-19) have affected every aspect of our lives. From the beginning of pandemic until now, a total of 676,609,955 confirmed cases of SARS-CoV-2 infection and a total of 6,881,955 deaths attributed to the virus have been recorded worldwide (https://coronavirus.jhu.edu/map.html, accessed on 18 January 2024). Unfortunately, after three years, despite the introduction of various vaccines, the virus is still as present in the human population as it was in 2020 [1]. In Puerto Rico, a Caribbean Island of 3.2 million people, the first confirmed case occurred in early March 2020, and by 21 September 2022, the island recorded 1,101,469 cases, confirmed by swab molecular tests, and 5823 deaths associated with COVID-19 (https://coronavirus.jhu.edu/region/us/puerto-rico, access on 17 November 2023). Government measures were characterized by national lockdowns together with contact tracing and isolation of diagnosed cases through passive surveillance of suspected cases [2].

Since the beginning of the COVID-19 pandemic, numerous quantitative/qualitative serological assays were developed across the globe as tools for detecting the presence of specific antibodies against SARS-CoV-2 [3,4,5,6]. As part of our contribution, our research group developed in-house serological assays to detect IgG antibodies in sera or plasma from individuals with COVID-19 in Puerto Rico [7]. Although serological approaches based on the detection of IgG cannot distinguish between acute and chronic infections, this type of test is useful for (i) the identification of individuals that have developed an immune response and could serve as plasma donors; (ii) facilitating contact tracing; (iii) determining the immune response dynamics in response to natural SARS-CoV-2 infections or mRNA vaccination; and (iv) determining the capacity to induce neutralizing antibodies against SARS-CoV-2 after mRNA vaccination in populations with concomitant inflammatory diseases [2,7,8,9].

Antibody production is a hallmark of the adaptive immune response and plays essential roles in neutralizing or eliminating microbial agents. Studies of the immune responses after a natural infection with SARS-CoV-2 have demonstrated that most symptomatic patients develop IgG antibodies within 2 weeks of symptom onset and the levels of these antibodies are dependent on the severity of the disease [10,11]. However, it has been demonstrated that asymptomatic patients also develop a humoral response to SARS-CoV-2 [12]. Most of the serological tests are based on the detection of IgM and/or IgG [13], even though IgA antibodies also play an important role in mucosal immunity and have an earlier onset than IgM during SARS-CoV-2 infections [13,14]. IgG is undoubtedly the most studied antibody during SARS-CoV-2 infections and several recent studies have demonstrated that this immunoglobulin remains at detectable levels in convalescent patients after several months of a natural infection [15,16]. The naturally induced IgG antibodies play a crucial role in facilitating the natural recovery of a majority of patients [17]. However, IgG exists in the form of four subclasses (IgG1, IgG2, IgG3, and IgG4), each with different properties [18]. The IgG profile in severe cases of COVID-19 has been previously characterized [19,20]. IgG1 and IgG3 are the dominant antibody isotypes elicited specifically against the virus spike (S) protein and receptor-binding domain (RBD) [21] during the acute phase of the infection. IgG1 has the ability to bind to Fc receptors on immune cells, causing antibody-dependent cytotoxicity (ADCC) and triggering complement-dependent cytotoxicity (CDC) [22]. IgG3 binds with high affinity to a variety of receptors (FcgRIIa, FcgRIIIa, and FcgRIIIb) on the surface of neutrophils, macrophages, and natural killer (NK) cells, and regulates the activity of these effector cells [23]. While it is reasonable to assume that these isotypes are present in non-hospitalized subjects, whether asymptomatic or with mild symptoms, limited knowledge exists regarding the distribution of the four IgG subclasses (G1, G2, G3, and G4) and their efficacy in neutralizing various variants of concern (VOCs) within this population during the primary infection. Furthermore, it is acknowledged that upon the administration of two doses of the SARS-CoV-2 mRNA-1273 (Moderna) vaccine, IgG1 and IgG3 are the predominant IgG subclasses [24]. However, the long-term development of all four IgG subclasses after the second dose with the Pfizer–BioNTech vaccine, and particularly after repeated vaccine doses, has been poorly studied. In this context, the characterization of the four IgG subclasses in naturally infected subjects compared to those that have received two or more vaccinations seems to be important considering that a large number of re-infections have been reported in several countries [25,26]. The characterization of IgG subclasses is also relevant due to the emergence of new SARS-CoV-2 variants, including Alpha, Delta, Omicron, and subsequent lineages [27], which may influence the retransmission, diagnosis, and prevention of infections.

The aim of the present study was to characterize the profile of the four anti-SARS-CoV-2 IgG subclasses (G1, G2, G3, and G4) produced after natural infection in a non-hospitalized COVID-19 Latino cohort with different durations of convalescence, and to compare this profile with those of convalescent or naïve subjects that received a full-course of an mRNA vaccine: Pfizer–BioNTech or Moderna (mRNA-1273). The study also aimed to compare the anti-SARS-CoV-2 IgG1, G2, G3, and G4 dynamics in immunocompetent and immunocompromised subjects that received multiple vaccinations with the Pfizer–BioNTech vaccine and correlate these antibody responses with the ability to neutralize the virus. The longitudinal evolution of the four anti-SARS-CoV-2 IgG subclasses in individuals with different immune states is highly relevant for estimating the long-term immune effects of vaccination or booster doses in the context of constant breakthrough variant infections.

## 2. Materials and Methods

### 2.1. Antigen and Reagents

We used commercially available recombinant SARS-CoV2 spike-1-RBD from GenScript (No. Z03483-1), which can bind with human ACE2 in functional ELISAs. This protein is produced in human cells with a predicted molecular weight of 30 kDa and >90% purity, as analyzed by SDS-PAGE (GenScript, Piscataway, NJ, USA). The study also employed disposable, high-bind, clear, flat-bottomed, polystyrene 96-well plates (Costar, Corning, MA, USA, No. 3361). For the secondary antibody in the IgG ELISA, we used a mouse anti-human IgG Fc conjugated with horseradish peroxidase (HRP) (GenScript, Catalog No. A01854), which is a mouse anti-human IgG Fc (50B4A9) monoclonal antibody that specifically reacts with human IgG and does not cross-react with IgG, IgA, or IgY antibodies from other animal species. For detecting the IgG antibody subclasses, we used anti-human IgG1 Fc (Southern Biotech 364 9054-05), anti-human IgG2 Fc (Southern Biotech #9060-05), anti-human IgG3 hinge (Southern Biotech 9210-05), and anti-human IgG4 Fc (Southern Biotech 9200-05). Carbonate–bicarbonate buffer with sodium azide (Sigma-Aldrich catalog No. 08058) and phosphate–citrate buffer (catalog No. P4809) with *o*-phenylenediamine hydrochloride (10 mg tablets) (Sigma-Aldrich, catalog No. P8287) were used for coating and as the buffer substrate, respectively. In the neutralization assay, we used a SARS-CoV-2 receptor-binding domain (RBD)–horseradish peroxidase (HRP) conjugate (RBD-HRP) from the following variants: wild-type (Genscript, Cat. No. Z03594), Alpha (B.1.1.7) (Genscript, Cat. No. Z03595), Delta (B.1.617.2) (Genscript, Cat. No. Z03614), and Omicron (B.1.1.529) (Genscript, Cat. No. Z03730).

### 2.2. Study Cohorts

The samples in this study were allotted to five different cohorts depending on the source. Cohort 1 included SARS-CoV-2-infected, non-hospitalized, non-vaccinated subjects. This cohort consisted of 85 deidentified samples (31 serum and 54 plasma samples) that had been collected during the pandemic from December 2019 to May 2020 (Table 1). Some of these samples were kindly donated by clinical laboratories and others were donated by blood banks serving the University of Puerto Rico Medical Sciences Campus (UPR-MSC) network, which received self-enrolled subjects for the purpose of donating plasma for the treatment of COVID-19 patients. The only information gathered from these donors was that they had not been hospitalized; some did not develop any clinical symptoms and their RT-qPCR-based positive diagnosis for SARS-CoV-2 was incidentally discovered during routine PCR testing of their samples. Other retrieved information collected from 69 of the 85 subjects was the date on which the confirmatory RT-qPCR was performed and the dates on which subsequent samples were collected. Thus, these 69 COVID-19 samples were allotted to three categories: 1–30 days, 31–60 days, and >60 days after infection, based on the time elapsed between the confirmatory RT-qPCR diagnosis and the date on which the subsequent sample was collected. The subjects were given the opportunity to ask the blood bank workers questions regarding their participation. Furthermore, the collected samples were handled using the standard blood donor protocols, along with the blood bank’s signed consent form, which also detailed the possibility that the samples would be used for research purposes.

Cohort 2 (previously infected individuals that received two doses of an mRNA vaccine: Pfizer–BioNTech or Modern-1273) consisted of serial samples from 12 subjects (7 females and 5 males) collected between October 2020 and September 2021. These individuals had been confirmed to have a SARS-CoV-2 infection between 30 and 150 days (median 75 days) prior to providing their baseline sample and receiving the first Pfizer–BioNTech or Moderna-1273 dose. From each of these subjects, three samples were collected: sample 1 (baseline) was collected prior to the administration of the first vaccine dose; sample 2 was collected at a median of 21.5 days after the second Pfizer–BioNTech or Moderna-1273 dose; and sample 3 was collected at a median of 96 days after the second Pfizer–BioNTech or Moderna-1273 dose (Table 2).

Cohort 3 (individuals with no previous SARS-CoV-2 infection and who received multiple mRNA vaccinations) was divided into two sub-cohorts. Cohort 3a comprised serial samples from 4 subjects (3 females and 1 male) that had no documented previous SARS-CoV-2 infection. These subjects were followed up for almost two years and received two doses of the Pfizer–BioNTech vaccine, a third dose (booster), and the bivalent vaccine. From each subject, six samples collected. Sample 1 was collected at baseline (prior to vaccination); sample 2 was collected at a median of 20 days after the second dose; sample 3 was collected at a median of 31 days after the third dose; sample 4 was collected at a median of 277.5 days (~9 months) after the third dose; and samples 5 and 6 were collected at a median of 30 and 180 days (6 months) after the bivalent vaccine, respectively. Cohort 3b included a single specimen from 8 individuals (4 females and 4 males) that received multiple vaccinations. These subjects had no previous documented SARS-CoV-2 infection prior to receiving the Pfizer–BioNTech vaccine. Among these individuals, four had received three vaccine doses, two subjects had received four doses, and two others had received a combination of three doses and the bivalent vaccine. Six of these subjects had a documented SARS-CoV-2 infection between 90 and 365 days (median: 240 days) following the third dose. The samples used in this study were collected between 351 and 723 days (median: 634 days or 1.7 years) after receiving the last vaccination (Table 2).

Cohort 4 consisted of samples from five individuals with inflammatory bowel disease (IBD) who had not been previously infected with SARS-CoV-2. These subjects were receiving treatment with immunomodulators and/or biologics and, at the time of the sample collection, they were in remission. Four samples from each subject were included in the present study: sample 1 was collected prior to vaccination (baseline); sample 2 was collected at a median of 17 days after the second dose; sample 3 was collected 60 days after the third dose; and sample 4 was collected at a median of 180 days after the third dose (Table 2).

Cohort 5 (pre-pandemic samples) consisted of 125 serum samples from deidentified adult donors, from which 78 were from subjects with an unknown health status and 47 were from subjects with other common respiratory viral infections or allergies affecting the Puerto Rican population (Table 1). These samples had been collected in 2012 and stored at the sample bank of the Immunology and Molecular Parasitology Laboratory of University of Puerto Rico Medical Sciences Campus (UPR-MSC). Samples from individuals with respiratory allergies or viral infections were collected in 2019 and banked at the Virology Laboratory of UPR-MSC or were kindly donated by the Center for Disease Control and Prevention (CDC) Dengue Branch, San Juan, PR. These samples included 13 specimens from subjects with a history of respiratory allergies, 5 from subjects with a Zika-IgM-positive diagnosis, 5 from subjects with a Dengue virus-IgM-positive diagnosis, 12 subjects with an RT-qPCR-base Influenza A/B-positive diagnosis, 6 from subjects with a Respiratory Syncytial Virus (RSV)-IgM-positive diagnosis, and 6 samples from individuals with a positive diagnosis for mycoplasma-IgM. Samples from Cohort 5 were stored at −80 °C and the aliquots tested had not been thawed prior to testing.

### 2.3. Ethical Statement

None of the samples analyzed in the present study were specifically collected for this study. They were kindly donated by our collaborators. Prior to receiving the samples, they were stripped of all identifiers so that the information could not be traced back to the individuals. The only information gathered from most of donors was the sex, age, and date on which the confirmatory swab molecular test was performed (Cohorts 1 and 2); and the type of vaccine administered, the date of the vaccinations, and the date of any breakthrough infections (if any) after vaccination (Cohorts 2, 3, and 4). The subjects from Cohorts 2 and 3 were all adults (>21 years old) and their samples were collected by the Virology Laboratory of the University of Puerto Rico-Medical Sciences Campus. These individuals were all volunteers participating in the IRB-approved clinical protocol “Molecular Basis an Epidemiology of Viral Infections circulating in Puerto Rico”, Pro0004333, which was approved by the Advarra IRB on 21 April 2020. The subjects from Cohort 4 were also adults (>21 years old) recruited at the University of Puerto Rico IBD Clinics and were all subjects diagnosed with Crohn’s disease (CD) or ulcerative colitis (UC) who were in remission and being treated with biologic and/or immunomodulatory therapy. An informed consent form and a study questionnaire also approved by the IRB was administered to all subjects (protocol approved by the IRB of the University of Puerto Rico Medical Sciences Campus, #1250121). The samples were collected by the University of Puerto Rico Gastroenterology Unit between April 2021 and July 2022 and were deidentified for the present study.

### 2.4. Detection of Anti-SARS-CoV-2 IgG Antibodies

The total titers of IgG antibodies to SARS-CoV-2 were measured using a pre-established in-house ELISA that was optimized through checkerboard titration, as previously described [28]. Briefly, 96-well plates were coated overnight with 100 µL/well of recombinant spike (S1)-RBD protein at a concentration of 2.5 µg/mL. The unbound spike-RBD was removed by washing thrice with 300 µL/well of phosphate-buffered saline containing 0.05% Tween-20 (PBST). Non-specific binding was blocked by adding 300 µL/well blocking buffer (5% skim milk in PBST) and incubating for 30 min at 37 °C. After the incubation, the blocking solution was removed using suction. The serum or plasma samples were diluted 1:100 in PBST and added in duplicate to the plate (100 µL/well). Wells containing the blocking buffer were used as blank wells. The plates were washed three times after incubation of 30 min at 37 °C. Then, the secondary antibody (anti-human IgG antibody peroxidase (1:10,000) diluted in blocking solution) was added to each well (100 µL/well) and incubated for 30 min at 37 °C. After another washing step, the peroxidase reaction was visualized by adding the substrate solution (100 µL/well of 0.1M citrate phosphate buffer, pH 5.0, containing 20 mg *o*-phenylenediamine hydrochloride and 30% H_2_O_2_). The reaction was incubated in the dark at room temperature for 15–20 min and stopped by adding 50 µL/well of 1N hydrochloride acid. Absorbance at 492 nm (OD_492_) was measured with a spectrophotometer. The OD_492_ values of blanks were subtracted from the corresponding OD_492_ values of each sample before the data analysis. As positive and negative controls, we used anti-SARS-CoV-2 IgG immunoglobulin purified with affinity chromatography from plasma donors with high titers of IgG, as previously described [7].

### 2.5. Detection of SARS-CoV-2 IgG Subclasses

To determine the levels of the IgG subclasses, we followed the same optimized protocol used for the in-house IgG ELISA described above but replacing the secondary antibody with either anti-IgG1-, anti-IgG2-, anti-IgG3-, or anti-IgG4-HRP diluted 1:3000 in PBST, as previously reported [3], which were incubated for 30 min at 37 °C. The substrate solution was 0.1 M citrate phosphate buffer, pH 5.0, containing 20 mg *o*-phenylenediamine hydrochloride and 30% H_2_O_2_, and the absorbance was read at 492 nm.

### 2.6. Neutralization Assay

To measure neutralizing activity, we used the cPass^TM^ SARS-CoV neutralization antibody detection kit (GenScript, Piscataway, NJ, USA) [29], which correlates perfectly with traditional PRNTs [7]. The assay was used to measure the inhibitory capability based on the ability of the antibodies to target the interaction between the host ACE2 receptor and viral receptor-binding domain (RBD). Briefly, samples (serum or plasma) were diluted in the sample dilution buffer according to the manufacturer’s instructions and incubated with either soluble SARS-CoV-2 RBD-HRP from the wild-type strain or the RBD-HRP from three variants of concern: Alpha (B.1.1.7), Delta (B.1.617.2), and Omicron (B.1.1.529). The mixtures were incubated for 30 min at 37 °C, which permitted the interaction and binding of antibodies with neutralizing capacity to the corresponding RBD-HRP conjugate. Following incubation, each reaction mixture was then added to a 96-well capture plate coated with human ACE-2 protein. Thus, RBD-HRP from each variant complexed with antibodies cannot bind to the coated ACE-2 protein and is then removed in a subsequent wash step. The reaction was developed using tetramethylbenzidine (TMB) followed by a stop solution, allowing the visualization of bound RBD-HRP to the ACE2 proteins. Since this is an inhibition assay, color intensity is inversely proportional to the number of neutralizing antibodies present in the samples. The data were interpreted by calculating the percent of inhibition of RBD-HRP binding as follows: Percentage of inhibition = (1-OD value of sample/OD value of background) × 100%. A percentage of inhibition ≥30% in a surrogate virus neutralization test (sVNT%) indicates an effective viral variant neutralization capacity.

### 2.7. Statistical Analysis

All antibody determinations were performed in duplicate and the results were reported as the mean absorbance for each determination, and each experiment was replicated twice. An ROC (receiving operating characteristic) curve was generated to establish the cut-off value of all assays using the EpiTools epidemiological calculator (http://epitools.ausvet.com.au (accessed on 9 November 2023)). The area under the curve (AUC), as a measure of the performance of our in-house ELISAs in discriminating between positive and negative results, was analyzed according to Hosmer et al. 2013 [30] as follows: totally random (AUC = 0.5); poor (0.5 < AUC < 0.7); acceptable (0.7 ≤ AUC < 0.8); excellent (0.8 ≤ AUC < 0.9); and outstanding (AUC ≥ 0.9). The calculation of positive and negative percent agreement (PPA and NPA) and overall rate of agreement (ORA) between the in-house ELISA method and the RT-qPCR method was determined using the method of Obermier et al., 2016 [31]. A Deming regression analysis [32] was performed on 10 paired plasma and serum specimens collected from the same individual to demonstrate the equivalence in the assay results for both specimens. To determine if there is a correlation between the absorbance values obtained for the IgG-subclasses with the sVNT% obtained against SARS-CoV-2 variants of concern, as well as between the sVNT% and the ELISA results, we calculated the Pearson correlation coefficient (with 95% CI) and Cohen’s Kappa coefficient (κ) [33,34], respectively. The κ values were categorized as follows: slight agreement (κ = 0.01 to 0.2); fair agreement (κ = 0.21 to 0.40); moderate agreement (κ = 0.41 to 0.60); substantial agreement (κ = 0.61 to 0.80); and almost perfect agreement (κ = 0.81 to 1.0) [35]. All statistical analyses were performed using GraphPad Prism 9.

## 3. Results and Discussion

The immunity against SARS-CoV-2 cannot be measured using a single parameter. It is molded by various factors including the genetic background of the population [36] and the manner in which our immune system responds to the evolution of new SARS-CoV-2 variants, each one with a greater immune evasion capability. Nowadays, a wide range of immune states exist in individuals who are unvaccinated: convalescing from a prior infection, recovering from one or more prior infections, infected and fully vaccinated, naïve to infection, vaccinated with or without booster injections, and those that have received multiple vaccinations and suffer from comorbidities. Although the humoral response does not drive the anti-viral response, the measurement of serum antibody levels, especially IgG, is globally accepted as a good marker for assessing such a wide variety of immune states in the populations exposed to COVID-19 and vaccinations [37]. This is the first study that describes the longitudinal evolution of the four IgG antibody subclasses in a Latin population with different immune status.

### 3.1. Assessment of In-House IgG and IgG Isotype ELISA Performance

**Figure 1:**
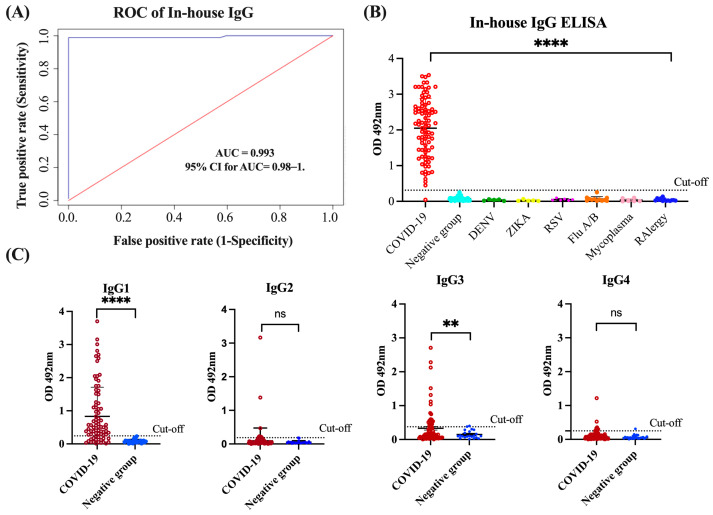
**Receiver Operating Characteristic (ROC) curve and OD distribution**. (**A**) ROC curve analysis of in-house anti-SARS-CoV-2 IgG ELISA performance including the are under curve (AUC) and 95% confidence interval 9CI). (**B**) OD distribution of sera/plasma from COVID-19 subjects, negative controls and from individuals with other respiratory or viral infections. (**C**) OD distribution of the four IgG subclasses of anti-SARS-CoV-2 IgG antibody detected in samples from COVID-19 subjects and negative control samples. **** *p* < 0.0001, ** *p* = 0.0025, ns = No significant differences.

In our in-house anti-SARS-CoV-2 IgG ELISA, we used the SARS-CoV-2 spike (S1) receptor-binding domain (RBD) as the antigen. Previous studies have demonstrated that the S1 domain is the most specific antigen for the diagnosis of COVID-19 while the RBD exhibits greater sensitivity, particularly in diagnosing patients with mild infections [38]. The N protein and S2 domain of SARS-CoV-2 may not be the optimal targets for diagnosing COVID-19 because of their high levels of cross-reactivity with the spike protein of SARS and MERS-CoV [39]. Therefore, we utilized the S1-RBD protein of SARS-CoV-2 as the target antigen for our in-house IgG ELISA. To establish the optimal positive cut-off for detecting the levels of anti-SARS-CoV-2 IgG antibody in sera/plasma of COVID-19 convalescent subjects, we created an ROC curve. For the positive control group, we used a cohort of 85 specimens (Cohort 1) from COVID-19 convalescent subjects that had been confirmed with RT-qPCR. These subjects had not been hospitalized, either because they had mild symptoms, were asymptomatic, or had been diagnosed through the active contact tracing program established by the Department of Health of the Commonwealth of Puerto Rico [2]. For the negative control group, we used a cohort of 125 specimens from healthy subjects or subjects that had been diagnosed with other respiratory or viral infections collected several years before the pandemic (Cohort 5). The ROC curve analysis revealed that the in-house IgG ELISA has outstanding diagnostic performance with an area under the curve (AUC) value of 0.993 (95% confidence interval (95% CI): 0.98–1) (Figure 1A). The OD distribution for the total IgG levels of the validated samples is shown in a scatter plot with the cut-off lines in Figure 1A. We found that the OD values 5-fold higher than the mean OD of the negative control (OD > 0.312) could be used as a positive threshold. Based on this threshold, 84 out of the 85 (98.8%) RT-qPCR-positive samples were identified as seropositive. Significant differences (*p* < 0.0001) were found between the mean OD values of the positive group and the negative group, and no cross-reactions were detected with samples from healthy subjects or those with respiratory or other viral infections (Figure 1B). These results correspond with a positive percentage agreement (PPA) between the RT-qPCR and the in-house IgG ELISA of 98.89%, a negative percentage agreement (NPA) of 100.00%, and an overall rate agreement (ORA) of 99.53% (Cohen’s κ = 0.990). Moreover, the Deming regression analysis revealed a linear correlation between the results from the serum and plasma specimens (*p* < 0.0001), demonstrating that the specimen type does not affect the in-house IgG ELISA results (Figure S1A,B). We also assessed the reproducibility of the assay by calculating the coefficient of variation (CV) of three different assays, with 30 repeats of the controls and selected negative and positive samples. The intra-assay and inter-assay reproducibility values were all lower than 10% (Figure S1B). Such an excellent diagnostic performance was further confirmed when the in-house IgG ELISA was validated with excellent results along with 27 other in-house ELISAs, 13 multiplex assays, and 12 different commercial serological methods developed across the globe as part of the serological science network (SeroNet) for COVID-19, which was established in October 2020 by the National Cancer Institute (NCI) [40].

The in-house ELISAs for detecting the IgG1, G2, G3, and G4 isotypes were developed on the same platform described above for total IgG. To establish the thresholds, we tested 85 samples from COVID-19 patients and a subset of 20 samples selected at random from Cohort 5 that included 10 samples from healthy subjects and 10 from subjects carrying other viral infections. We found that OD values that were 4.56-fold (OD > 0.242), 3.44-fold (OD > 0.186), 3.31-fold (OD > 0.375), and 4.1-fold (OD > 0.250) higher than the mean OD of the negative control could be used as positive thresholds for determining the levels of IgG1, IgG2, IgG3, and IgG4, respectively (Figure 1C). Based on these thresholds, IgG1 was detected in 62 of the 85 samples (72.94%) of convalescent COVID-19 patients and IgG3 was detected in 25 of the 85 samples (29.41%), whereas IgG2 and IgG4 were detected in only 7 (8.23%) and 8 (9.41%) of the 85 samples, respectively. IgG1 was not only the most frequently detected isotype, but also the one with the highest levels, as indicated by the OD values, which ranged between 0.29 and 3.156 (mean OD = 1.115 ± 0.86). The OD values for IgG3 ranged between 0.379 and 2.28 (mean OD = 0.881 ± 0.63). Hence, the average OD for IgG1 was 1.27-fold higher than that of IgG3, which was significantly different (*p* < 0.0001). These results are consistent with the notion that IgG1 and IgG3 isotypes are typically elicited in response to viral infections [18,41] and can be considered prominent markers of the late stage after the primary SARS-CoV-2 infection. Therefore, IgG1 and IgG3 are the main contributors to the IgG titers commonly reported in the serological assays for COVID-19 (Table S1).

**Figure 2:**
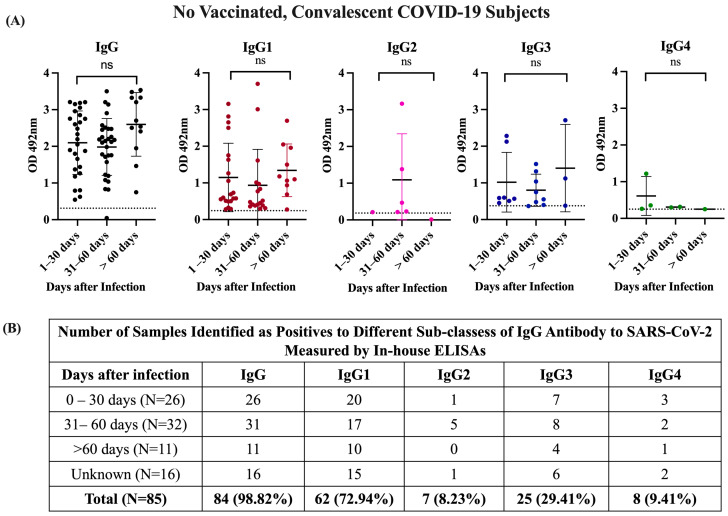
**OD distribution of specimens and number of positive samples from unvaccinated COVID-19 convalescent subjects.** (**A**) OD distribution of samples from unvaccinated, convalescent COVID-19 subjects tested using in-house IgG and IgG subclass ELISAs, which were allotted into three groups (1–30 days, 31–60 days, and >60 days since the date of confirmatory SARS-CoV-2 RT-PCR testing). There were no significant differences (ns) between OD values of the different IgG isotypes regarding to the time of infection. (**B**) Number of seropositive samples for each IgG subclass according to the days after infection.

Moreover, the observation that most specimens collected between 0 and 30 days after infection (79.6%) or at >60 days after infection (90.9%) were positive for IgG1 or IgG3 (Figure 2) indicates that convalescent subjects develop high levels of these antibody isotypes early in the course of infection and they remain at detectable levels for several months after recovery. This is an important finding since none of the subjects in this study required hospitalization and most existing knowledge regarding the antibody response against SARS-CoV-2 has been collected from hospitalized individuals with various grades of COVID-19 disease severity, where the magnitude of the antibody response to a SARS-CoV-2 infection correlated with the severity of the disease [42].

In contrast, aside from two samples that were found to be highly immunoreactive for IgG2 (OD = 1.383 and 3.166, respectively), the other five positive samples showed very low OD values, which ranged between 0.211 and 0.469 (mean OD = 0.267 ± 0.1). For IgG4, only one specimen had a high OD value (OD = 1.22), and the other seven seropositive samples had OD values barely over the threshold and which ranged between 0.253 and 0.542 (mean OD = 0.336 ± 0.0.09) (Table S1). Thus, not only were IgG2 and IgG4 less frequently detected, but they are also the less immunoreactive isotypes against S1-RBD of SARS-CoV-2, which is consistent with the reports from other authors [41]. The IgG2 and IgG4 antibody isotypes are not commonly part of the antibody response to viral infections. IgG2 plays a role in the response to bacterial infections, whereas IgG4 has a role in the response to allergens, helminth infections, or therapeutically administered proteins [43]. The IgG isotype patterns observed in the present study seem to be slightly different from those reported for symptomatic patients who experienced different degrees of disease severity. In such patients, the presence of the four IgG isotypes was detected during acute infections, with IgG3 being the isotype most associated with the initial phase and severity of the disease, while IgG1 and IgG2 were the isotypes most associated with an upsurge in the late stage of the disease and with the weakest correlation with disease severity [42].

### 3.2. The Antibody Response in Unvaccinated Convalescent COVID-19 Subjects Is Dominated by IgG1 and IgG3 Isotypes, Which Neutralize the Wild-Type Strain and the Alpha and Delta VOCs but Are Poorly Effective against Omicron

Once the IgG isotype profile in the cohort of convalescent, unvaccinated, COVID-19 subjects (Cohort 1) was characterized, we used a surrogate virus neutralization assay to determine the neutralizing activity of the specimens from this cohort against wild-type SARS-CoV-2 and the VOCs Alpha (lineage B.1.1.7), Delta (lineage B.1.1617.2), and Omicron (lineage B.1.1.529), which were broadly circulated and predominant in the Puerto Rican population [44]. We found that 83 of the 84 seropositive specimens (98.8%) had a virus neutralizing percentage (sVNT%) against the wild-type (WT) strain that ranged between 33 and 97% (median = 82%). One seronegative specimen and another seropositive specimen for IgG failed to show a neutralizing activity. The seronegative specimen, which was also negative for IgM and the IgG isotypes, had been collected ~38 days after the positive RT-qPCR result was obtained (Table S1). Presumably, this subject harbored a low viral load, leading to the immune system clearing the infection prior to mounting an appropriate immune response. This circumstance could account for the difficulties encountered in detecting IgG antibodies in this specimen. However, it is possible that the RT-qPCR result could be a false positive, since several molecular assays have been developed with high specificity and low limits of detection, suggesting that the test could have detected minimal amounts of the virus, leading to a false positive result [45,46,47,48]. Interestingly, 63 of the 85 specimens (74.11%) showed a detectable sVNT% against the Alpha variant in the range of 30 to 93% (median = 58.0%), and 73 of the 85 samples (85.9%) showed an sVNT% value in the range of 32 to 90% (median = 64%) against the Delta variant. In contrast, the sVNT% against Omicron was low, being detected only in 3 of the 85 specimens (3.53%), with an sVNT% value barely over the threshold (31 to 41%, median = 31) (Table S2).

**Figure 3:**
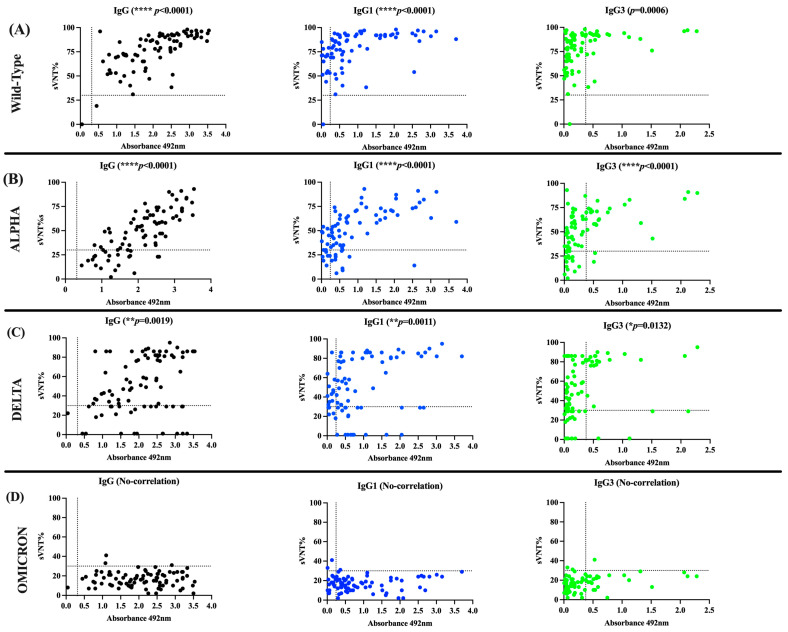
**Correlation between the levels of IgG subclasses and the neutralizing activity.** The OD values obtained for each IgG subclass was correlated with the surrogate virus neutralization percentage (sVNT%) obtained against (**A**) the wild type of strain and the VOCs (**B**) Alpha, (**C**) Delta, and (**D**) Omicron.

When the performance of the in-house IgG ELISA was compared with the neutralization assay in their capacity to detect seropositive specimens with detectable sVNT% values, a substantial agreement (98.87%, κappa value = 0.6615) was found for the WT strain, a slight agreement was found for the Alpha (75.29%, κappa = 0.0659) and Delta (69.41%, κappa = 0.0498) variants, and no agreement was found for the Omicron variant. We also found a slight agreement between the sVNT% values against the WT strain and the number of seropositive samples in the IgG1 ELISA (76.47%, κappa = 0.0502), a fair agreement against Alpha (72.94%, κappa = 0.2608), and no agreement against Delta or Omicron. Similarly, the IgG3 ELISA showed a slight agreement with the sVNT% values against the wild-type strain (30.58%, κappa = 0.0187), Alpha (48.23%, κappa = 0.1515), or Delta (50.58%, κappa = 0.1471), but no agreement with Omicron (Table 3). Consistent with these results, the magnitude of the total IgG, IgG1, and IgG3 levels, measured as average OD values above the established threshold for each isotype, correlated positively with the percentages of neutralization (sVNT%) against WT (*p* < 0.001, IgG and IgG1; *p* = 0.0006, IgG3), Alpha (*p* < 0.0001, IgG, IgG1, and IgG3), and Delta variants (*p* = 0.0019, IgG; *p* = 0.011, IgG1; *p* = 0.0132, IgG3) (Figure 3), and did not correlate with the number of seropositive samples or the levels of IgG2 or IgG4 for any of SARS-CoV-2 VOCs analyzed.

The observation that most samples from COVID-19 convalescent subjects exhibited remarkably high neutralizing percentages against the WT strain was expected. This is attributed to the fact that these specimens were collected at the beginning of the pandemic at the time that a wide diversity of lineage B.1.x variants, which emerged from the original Wuhan strain, were circulating in Puerto Rico [44]. Because the Alpha and Delta VOCs, which emerged and subsequently accumulated a relatively small number of mutations in their genome (especially in the spike protein, enhancing their fitness and pathogenicity [49,50]), it was not surprising that the antibodies elicited against the original strain could still partially neutralize these variants as well. In our study, these antibodies showed a median of 54% effectiveness against Alpha and a median of 65% effectiveness against Delta. However, the antibodies elicited against the original strains became completely ineffective against Omicron, which is the most heavily mutated VOC [51] that replaced Delta and other variants in circulation.

### 3.3. The Antibody Response in Previously Infected Subjects That Received Two Doses of the Pfizer–BioNTech or Moderna-1273 Vaccine Is Dominated by the IgG1 Isotype Which Has Potent Neutralizing Activity against the Alpha, Delta, and Omicron VOCs

The Pfizer and Moderna vaccines contain synthetic mRNA molecules with the coding sequence necessary to build the SARS-CoV-2 spike protein encased in a lipidic nanoparticle that allows the delivery of the mRNA to cells. Thus, this protein can be synthesized inside the host cell, mimicking a natural infection with SARS-CoV-2 [52]. To determine if the administration of two doses of an mRNA vaccine could modify the IgG subclass profile established by a previous natural infection, we selected a cohort of 12 convalescent subjects who received a full course of two doses of the Pfizer–BioNTech (*n* = 8) or Moderna-1273 (*n* = 4) vaccine (Cohort 2). There was an interval of three to four weeks between the two doses. From each subject, we analyzed a sample collected at baseline (sample 1) and two subsequent samples collected after a mean of 21.5 days (sample 2) and 96 days (sample 3) after the second dose. At baseline, all specimens but one had detectable levels of IgG antibodies. Considering that all these subjects had a single documented previous infection at a mean of 150 days (5 months) prior to receiving the first dose of the vaccine, it is plausible that the antibody response to the natural infection was long-lasting and did not wane as fast as has been reported by other researchers [53,54].

**Figure 4:**
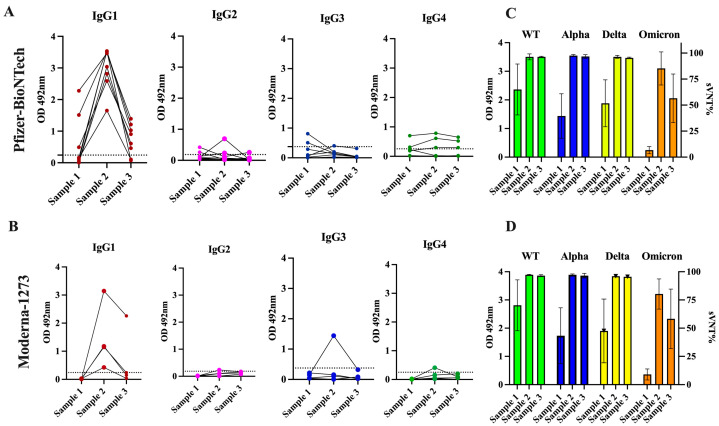
**Levels of IgG subclasses and neutralizing capacity in serial samples from previously infected subjects that received two doses of mRNA vaccine.** (**A**) The OD distribution of serial samples collected from previously infected subjects that received two doses of the Pfizer-BioNTech (*n* = 6) or (**B**) Moderna-1273 (*n* = 6) vaccines are showed. Sample 1 was collected before vaccination (baseline0, sample 2 was collected at a median of 21.5 days after the second dose, and sample 3 was collected at a median of 96 days after the second dose. Black dashed line represents the positive cut-off value for each in-house IgG subclass ELISA. (**C**) The average OD values for each sample from subjects that received the Pfizer-BioNTech or Moderna-1273 vaccine associated with (**D**) the corresponding average surrogate virus neutralization percentage (sVNT%) are showed.

At baseline, a total of three specimens (25.0%) had detectable levels of IgG1, and two specimens (16.6%) had IgG3 levels that ranged between 0.49 and 2.281 (mean OD = 1.427 ± 0.733) and between 0.509 and 0.813 (mean OD = 0.661 ± 0.152), respectively. IgG2 and IgG4 were only detected in a single specimen at very low levels. It was interesting to find that the antibody response detected after the second dose was exclusively dominated by the IgG1 isotype in all specimens, with OD values ranging between 1.651 and 3.536 (mean OD = 3.017 ± 0.619) in those vaccinated with the Pfizer–BioNTech vaccine and between 0.426 and 3.144 (average OD = 1.475 ± 1.00) in the subjects vaccinated with the Moderna-1273 vaccine. A single subject who received the Pfizer vaccine had detectable levels of IgG2, while another subject who received the Moderna vaccine had detectable levels of IgG3. However, at a mean of 96 days following the second dose (sample 3), the levels of IgG1 were undetectable in two subjects who received the Pfizer–BioNTech vaccine and in three subjects who received the Moderna-1273 vaccine. In the other subjects, the levels of IgG1 dropped to an average of OD = 0.723 ± 0.460 or OD = 0.669 ± 0.92 for those vaccinated with the Pfizer or Moderna vaccine, respectively. The levels of IgG4 were markedly low or undetectable during the entire follow-up period (Figure 4 and Table S3).

As we reported before, most specimens (91.66%) collected at baseline from these 12 subjects showed detectable neutralization percentages (sVNT%) against WT (average of 66.75% ± 23.96), 58.33% showed detectable sVNT% values against Alpha (42.33% ± 22.27), 83.3% showed these values against Delta (50.33% ± 24.69), and no specimens had detectable sVNT% values against Omicron. After receiving the second dose of a vaccine, the duration of neutralizing activity was similar for subjects that received the Pfizer or Moderna vaccine. In both groups, the sVNT% increased notably at >95% against all variants tested, including Omicron. However, about 96 days following the second dose, these levels significantly declined against Omicron (average 57.58% ± 23.44) in the subjects, regardless of the vaccine the subject received (Figure 4). No subjects reported having breakthrough infections during the follow-up period. It is possible that the natural immunity boosted by two doses of the mRNA vaccines contributed to preventing reinfections, although the antibodies declined over the same period, which is consistent with other studies [55]. A case–control study performed in Qatar found that natural infections were 90.2% effective (95% CI 60.2–97.6) in preventing reinfections with the Alpha variant, 85.7% effective against Beta (95% CI 75.8–91.7%), and 92% effective against Delta (95% CI 87.9–94.7), but were notably less effective against Omicron (56%, 95% CI 50.6–60.9) [56]. The observation that, in some subjects, the levels of antibodies declined faster than the neutralizing activity (Figure 4), reinforces the notion that the function of the antibodies measured as their capacity to bind to the RBD-spike protein and prevent the entry of the SARS-CoV-2 virus into cells is more reliable than the quantity of antibodies produced (measured as average OD) to monitor the antibody response against infections and vaccinations [7]. The observation that the durability of the neutralizing capacity induced by both vaccines against the Omicron variant declines faster is also consistent with other studies [57]. The Pfizer–BioNTech and Moderna-1273 vaccines were designed against the original SARS-CoV-2 strain and these vaccines produce antibodies with a substantially reduced ability to recognize and block the entry of the Omicron spike protein, which harbors numerous mutations [58].

### 3.4. Class Switch toward IgG4 Occurs in Subjects That Receive Multiple Doses of Pfizer–BioNTech Vaccine, Which Is Sustained over the Time

**Figure 5:**
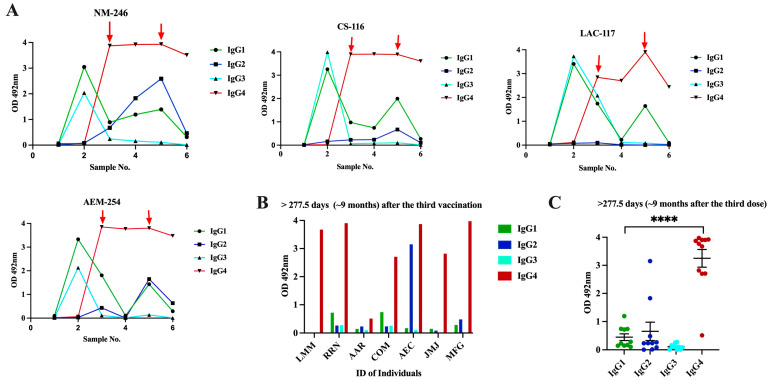
**Changes in IgG subclass levels in naive subjects that received multiple vaccinations with the Pfizer–BioNTech vaccine show a switch toward the IgG4 subclass.** (**A**) Six samples were collected from four (*n* = 4) naive subjects that received multiple vaccinations with the Pfizer–BioNTech vaccine (Cohort 3a). Sample 1 was collected at baseline, sample 2 was collected at a median of 20 days after the second dose, sample 3 was collected at a median of 31 days after the third dose, sample 4 was collected at a median of 255 days after the third dose, sample 5 was collected at a median of 30 days after the bivalent vaccine, and sample 6 was collected at a median of 180 days after the bivalent vaccine. Red arrows indicate the time at which the third dose or bivalent vaccine was administered to each subject. (**B**) A single sample from eight (*n* = 8) subjects (Cohort 3b), who received three doses of the Pfizer–BioNTech vaccine, was collected at a median of 277.5 days (~9 months) following the third Pfizer–BioNTech dose and for the levels of the IgG subclasses were measured. (**C**) OD distribution of each IgG subclass in the samples of Cohort 3b clearly demonstrate the dominance and durability of IgG4 response in subjects that received multiple doses of the Pfizer–BioNTech vaccine. **** *p* < 0.0001.

Because the neutralizing protection offered by the existing immunization schedule against the Omicron lineage is minimal [59], the Pfizer–BioNTech and Moderna-1273 vaccines were updated to include a monovalent (single) component that corresponds to the Omicron variant XBB.1.5. Furthermore, the U.S. Food and Drug Administration (FDA) authorized Moderna and Pfizer–BioNTech to use a single bivalent vaccine as a booster, which contains two mRNA components of the SARS-CoV-2 virus, one from the original strain and the another from a common component found in the BA.4 and BA.5 lineages of the Omicron variant (https://www.fda.gov/news-events/press-announcements/coronavirus-covid-19-update-fda-authorizes-moderna-pfizer-biontech-bivalent-covid-19-vaccines-use) (accessed on 19 December 2023). To determine whether the administration of multiple vaccinations could modify the profile of antibody isotypes induced by the initial two doses of the Pfizer–BioNTech vaccine, we followed up with a small group of four subjects (Cohort 3a) for almost two years and collected samples at different time points following the second and third doses and the administration of a bivalent vaccine. Before receiving the first dose, these subjects had not been previously exposed to SARS-CoV-2. As was expected, at baseline, every subject had detectable anti-SARS-CoV-2 antibodies (sample 1). The initial two vaccinations were administered with a three-week interval and the third vaccination (booster dose) was administered about eight months after the second dose. About 12 months after the booster dose, all subjects received the bivalent vaccine. As described before, IgG1 (average OD = 3.26 ± 0.135) and IgG3 (average OD = 2.97 ± 0.889) were the dominant isotypes shortly after the second dose (sample 2), with an absence of IgG2 and IgG4 (Figure 5A, Table S4). Our results are consistent with reports from others, which indicated that IgG1 and IgG3 specific to the spike protein of SARS-CoV-2 and its receptor-binding domain (RBD) are the predominant antibody subclasses [21] in individuals who are both infected and vaccinated. The Pfizer and Moderna vaccines contain synthetic mRNA molecules with the coding sequence necessary to build the SARS-CoV-2 spike protein encased in a lipidic nanoparticle that is delivered to host cells. This allows the protein to be synthesized within the host cell, mimicking a natural infection with SARS-CoV-2 [52]. Both IgG1 and IgG3 are typically elicited in response to viral infections [18].

**Figure 6:**
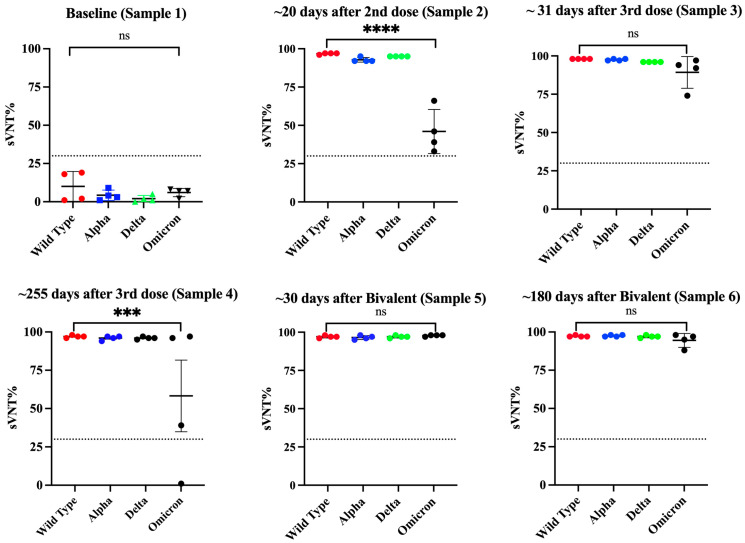
**Changes in the neutralizing capacity in specimens from naive subjects that received multiple vaccinations with the Pfizer–BioNTech vaccine.** Serial samples were collected from four naive subjects (*n* = 4) that received multiple vaccinations and tested using a surrogate neutralization assay to quantify the viral neutralization percentage (sVNT%) against the wild-type strain and the variants of concern (VOCs) Alpha, Delta, and Omicron. Sample 1 was collected at baseline, sample 2 was collected at a median of 20 days after the second dose, sample 3 was collected at a median of 31 days after the third dose, sample 4 was collected at a median of 255 days after the third dose, sample 5 was collected at a median of 30 days after the bivalent vaccine, and sample 6 was collected at a median of 180 days after the bivalent vaccine. Black dashed line represents the positive cut-off for the sVNT% (>30%). *** *p* = 0.0002, **** *p* = 0.0001. ns = No significant differences.

Of note, after the second vaccination, all samples showed very high sVNT% values (>95%) against WT and the Alpha and Delta variants and reduced sVNT% (46.0%) against Omicron (Figure 6). However, an intriguing observation was that the specimens of these subjects had 1.85-fold less neutralization capacity against Omicron than specimens from Cohort 2 (previously infected individuals fully vaccinated with Pfizer), which, at a similar time point after the second dose (~20 days), had an average sVNT% of 85.25%, and these differences were statistically significant (*p* = 0.0028). Moreover, the observation that the subjects of Cohort 2 at a mean of 96 days after the second dose still had 1.24-fold higher sVNT% (average 52.2%) against Omicron than Cohort 3a at 20 days indicates that the protection induced by the Pfizer–BioNTech vaccine wanes faster than the protection induced by a combination of natural infection plus vaccination. Interestingly, at a mean of 31 days after receiving the third dose (sample 3), IgG1 and IgG3 were no longer the dominant antibody isotypes. The IgG1 levels declined to an average of OD = 1.35 ± 0.422, which was 2.41-fold lower than those detected after the second dose, whereas IgG3 was undetectable in most of the subjects. The IgG1 levels continued declining and, at a mean of 277.5 days (~9 months) after the third dose (sample 4), they were barely detectable in two subjects (average OD = 0.565 ± 0.433). Intriguingly, after administering the third dose (a mean of 34 days), the IgG4 subclass, considered as an anti-inflammatory antibody [60], reached levels notably higher than the lower limit of quantification in the sera of all vaccinees (average OD = 3.615 ± 0.445) and remained at almost identical levels (average OD = 3.574 ± 0.506) at the subsequent sampling that occurred about 9 months later (Figure 5A). The third dose also elicited detectable levels of IgG2 in three subjects (75%) at low levels (average OD = 0.356 ± 0.216) around 30 days after being administered, but these levels became undetectable thereafter. The third dose also boosted the sVNT% against Omicron to a range between 74 and 97% (median 93%), but these levels dropped again to an average of 67.5% around 9 months after the third dose (Figure 6).

It was interesting that during the time between the second and the third doses, none of the subjects reported being naturally infected. However, about 3 to 4 months after receiving the third dose, two subjects reported having a breakthrough SARS-CoV-2 variant infection. This infection did not appear to have any effect on the IgG4 levels as judged by the lack of fluctuations in the OD values for IgG4 in these subjects. To determine if a subsequent immunization with the bivalent vaccine could modify IgG4 levels, two additional samples at 30 and 180 days following the bivalent vaccination were analyzed. Our results show that IgG4 levels were boosted in only one subject after the administration of the bivalent vaccine, while in the other subjects, the IgG4 levels remained unaltered. In turn, the bivalent injection increased the levels of IgG1 in all subjects (average OD = 1.615 ± 0.239), as well as the IgG2 levels in most of individuals (75%) (average OD = 1.636 ± 0.782) (sample 5), although both isotypes dropped to background levels or were undetectable during the subsequent follow-up course (sample 6). The IgG3 isotype was not detected in any of specimens after the third dose or bivalent vaccine. After the administration of the bivalent vaccine, the neutralizing percentages against all VOCs, including Omicron, were also boosted; however, in contrast to the IgG1 levels, the neutralizing percentages remained high, in a range between 88 and 98% (median = 96%), at 180 days after receiving the bivalent vaccine (Figure 6).

Because our cohort of multi-vaccinated subjects was small and it was not possible to obtain samples from these subjects over a longer period, we decided to analyze an independent cohort to examine the contribution of IgG4 antibodies to the long-lived antibody pool after the third immunization. The new cohort comprised samples from eight subjects (Cohort 3b) that had received the primary two doses of the Pfizer–BioNTech vaccine and a booster dose. A single specimen from each of these subjects was collected between 12 and 24 months after receiving the third dose or bivalent vaccine (Table 2). None of these individuals had a documented SARS-CoV-2 infection prior to receiving the initial two Pfizer–BioNTech doses; however, six of them had at least one documented breakthrough infection, occurring between 30 and 60 days after the third dose. Intriguingly, despite the specimens from these subjects having been collected long after the booster dose or bivalent vaccine, seven of the eight specimens had high levels of IgG4 with OD values ranging from 2.709 to 3.975 (average OD = 3.557 ± 0.510). Low to moderate levels of IgG1 (from 0.286 to 0.738; average OD = 0.58 ± 0.208) were also detected in three of the subjects. The IgG2 isotype was only detected in two subjects (average OD = 3.153 and 0.482, respectively) (Figure 5B). When the data of all specimens from Cohorts 3a and 3b were collectively analyzed, it was confirmed that all subjects with multiple Pfizer–BioNTech vaccinations had significantly higher levels of IgG4 compared to IgG1, IgG2, and IgG3 after ~30 days (*p* = 0.0003) and at a mean of 18 months (*p* < 0.0001) following the booster dose (Figure 5C), which confirms that a class switch toward IgG4 was induced after the third dose of vaccine and remained at high levels for a long period of time. No statistical differences were found between the IgG4 values of subjects that had breakthrough infections after the third dose compared to those who did not have documented infections with SARS-CoV-2. Because our cohort of subjects that received multiple vaccinations only comprised subjects that received the Pfizer–BioNTech vaccine, we were unable to determine whether the IgG4 subclass switch also occurs in subjects that receive multiple doses of the Moderna vaccine. However, this would not be unlikely, because both vaccines contain synthetic mRNA molecules that induce immune responses through the same mechanism, and we did not find differences regarding the IgG profiles associated with the type of vaccine in the cohort that received two doses of Pfizer–BioNTech or Moderna-1273.

Although we were unable to obtain samples from subjects that received multiple doses of Moderna-1273, another research group has documented this switch in individuals that received both vaccines [61]. However, in contrast to this study, this other group of researchers found that the switch in class toward IgG4 occurred some months after the second dose in approximately 0.04% of the subjects [61]. Interestingly, the samples of all the subjects in our study also showed very high sVNT% values against all VOCs, including Omicron, ranging between 95 and 98%, which was concurrent with the high levels of IgG4 in the same samples. The presence of IgG4 in these samples is atypical because it is not considered an anti-viral IgG subclass and IgG4 is not expected to play a role in preventing the entry of viruses into cells. Thus, it is possible to speculate that the observed neutralizing activity in these samples can be attributed to the IgG1 isotype, which was found at high levels following every booster, or attributed to IgA [62] or IgM [63], which can also have a role in neutralizing the virus but were not measured in the present study.

### 3.5. Switch in Class toward IgG4 Is Also Observed in Subjects with Inflammatory Bowel Disease and Received Multiple Vaccinations

**Figure 7:**
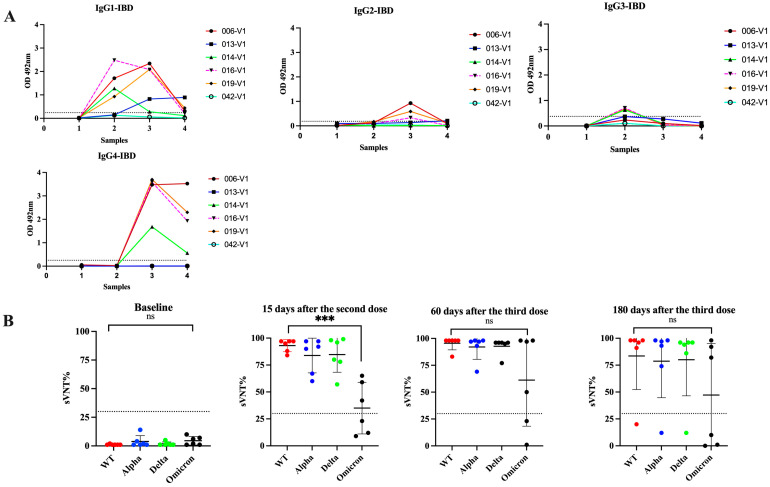
**Changes in IgG subclasses in immunocompromised subjects that received multiple vaccinations with the Pfizer–BioNTech vaccine.** Four samples were collected from six subjects (*n* = 6) with inflammatory bowel disease (IBD), who received standard treatment with immunomodulators or biologics. Sample 1 was collected at baseline, sample 2 was collected at a median of 15 days after the second dose, sample 3 was collected at a median of 60 days after the third dose, and sample 4 was collected at a median of 180 days after the third dose (Panel **A**). The neutralizing capacity of these samples, measured as the surrogate virus neutralization percentages (sVNT%) against the wild-type strain (WT) and the VOCs Alpha, Delta, and Omicron, is showed in (Panel **B**). *** *p* = 0.0002. ns = No significant differences.

Treatment with immunomodulators and/or biologic agents is standard in the management of moderate to severe IBD to achieve disease control and maintain remission. These medications impact the immune system and make them vulnerable to several microbial infections that require inflammatory responses for protection [64]. These treatments also reduce the protective immune response to vaccines such as hepatitis A/B, pneumococcus, and influenza [65]. A previous study of IBD patients in Puerto Rico revealed that although two doses of a COVID-19 mRNA vaccine induce seroconversion and neutralizing activity against several VOCs, these responses are mostly ineffective against Omicron, which does not seem to change after administering a booster shot [8]. In the present study, we tested serial samples from five IBD subjects that received multiple Pfizer–BioNTech doses (Cohort 4) to ascertain if the antibody class switch to IgG4 after the third dose also occurs in these subjects. The booster injection was given to these individuals about 6 months after the second dose. Our results showed that 15 days following the second dose of the Pfizer vaccine, four subjects (80.0%) elicited antibody responses to the vaccine similar to those observed in immunocompetent subjects with an average IgG1 subclass OD = 1.599 ± 0.579 and undetectable levels of IgG2, IgG3, or IgG4 in all specimens. After the third dose (30 days), IgG4 became the most prevalent antibody isotype (average OD = 3.11 ± 0.828) and remained at high levels for 180 days after the booster shot (average OD = 2.078 ± 1.056) (Figure 7). The only difference between the IBD group compared to the immunocompetent group is that at 180 days following the booster shot, the IBD group had average IgG4 levels 1.45-fold lower than those observed at 30 days after the third dose, suggesting a tendency to decline faster. In contrast, the IBD cohort showed low sVNT% values (average 56.4%) against Omicron, similar to the subjects of Cohort 3a, indicating that the immune responses elicited after the booster dose are ineffective against breakthrough infections in both groups.

The finding of a class switch toward IgG4 in both immunocompetent and immunocompromised subjects occurring after the third dose was the most striking finding of this study. It is not clear whether the consistent switch to this antibody isotype could be beneficious or detrimental for the Latino population that received multiple vaccinations. Interestingly, studies have reported that mRNA vaccines, and not those based on adenoviruses such as the AstraZeneca vaccine, are those inducing long-lasting IgG4 responses [61], although the reasons for this are still not very well understood. IgG4 is considered an unusual antibody because it exhibits a poor ability to destroy infected cells through the activation of the complement system [66,67].

IgG4 is typically induced during helminth infections and allergic diseases where this antibody subclass plays a protective role [61]. However, in other circumstances, a high level of IgG4 in the serum is considered pathogenic because it could trigger an autoimmune disease [68], cancer [69,70], or many other illnesses such as lymphadenopathy [71], interstitial pneumonitis [72], and aortic aneurism [73]. Hence, it has been proposed that instead of being beneficial, repeated vaccinations tend to induce immunological tolerance. This could occur because the amount of spike protein produced in response to repeated mRNA administration is too high and lasts too long in the body. The continuous exposure of T-cells to such a large amount of spike protein could desensitize the CD4+ and CD8+ cells, making them lose their capacity to proliferate and, consequently, lose their capacity to respond appropriately to re-infections with SARS-CoV-2 variants [74]. If this happens, the immune system could become exhausted, leading to autoimmunity [74]. Therefore, it is not unlikely that subjects who have received booster injections and who are re-exposed to the virus may suffer a more severe COVID-19 disease. Subjects with IBD could concurrently suffer from exacerbated intestinal inflammation. The induction of immunological tolerance by repeated vaccinations could perhaps explain the large number of deaths occurring in vaccinated people who received a third dose compared with unvaccinated individuals in some European countries [75,76,77]. These negative outcomes may be cumulative and manifest several years later. It is likely that aging people or immunodeficient individuals would be the most affected, which, paradoxically, are the populations that are more vulnerable to SARS-CoV-2 infection and are constantly encouraged to get vaccinated periodically.

## 4. Limitations of the Study

The main limitation of the present study was the small size of the cohorts comprising vaccinated individuals with or without booster injections, which restricts making conclusions that can be statistically validated using a power analysis. Considering that at the present time, most people around the world, and especially in Puerto Rico, have received at least a single dose of a SARS-CoV-2 vaccine, have been infected with SARS-CoV-2, or both, the possibility of obtaining larger Latino cohorts for each of the immune states mentioned above could be a formidable challenge. Despite this limitation, we consider the findings of this study to be valuable. Other limitations were the lack of samples from Cohort 3b, which corresponds to baseline or shorter time points before receiving the third dose. This prevented us from determining the exact time point at which the IgG4 levels began to rise in these subjects. We also could not add a group of people who had not been vaccinated and who had suffered from repeated infections. It would be interesting to investigate if repeated infections with several variants or subvariants could also induce the class switch to IgG4.

## 5. Conclusions

The most notable findings from this study can be summarized as follows: (1) COVID-19 convalescent subjects that were not hospitalized developed high and long-lasting antibody responses. (2) IgG1 and IgG3 are the most prevalent antibody subclasses in the SARS-CoV-2-infected population, whereas IgG1 is the most prevalent subclass after vaccination. (3) Individuals that first had a primary SARS-CoV-2 infection and later received two doses of an mRNA vaccine exhibited a more robust capacity to neutralize Omicron than individuals who were not infected and received two doses of the Pfizer–BioNTech vaccine, which reinforces the advantage of natural immunity combined with induced immunity. (4) A class switch towards the “anti-inflammatory” antibody isotype IgG4 is induced a few weeks after the booster dose with the Pfizer–BioNTech vaccine. The levels of IgG4 remain at high levels for a long period and seem to not be affected either by a subsequent booster dose (e.g., bivalent vaccine) or by breakthrough infections. (5) Immunocompromised subjects, such as those with IBDs receiving treatment with immunomodulators, also induce IgG4 after the third dose, although these antibody levels have a limited effect on their immune system’s neutralizing capacity. Further studies are needed to elucidate the mechanisms that underlie this class switch and determine its potential negative effect on people with comorbidities or immunosuppression, or who are at an older age. At the present time, recognizing that the mRNA vaccines do not prevent reinfections [59], that all Omicron subvariants that are dominant worldwide have been shown to be less pathogenic [78,79], and that there are potential negative effects of multiple boosters on the immune system, revising the continuous booster vaccination guidelines should be considered.

## Figures and Tables

**Table 1:** Characteristics of specimens from SARS-CoV-2 infected, no-hospitalized, no vaccinated and pre-pandemic cohorts used in the study.

**Cohort-1: SARS-CoV-2 infected; convalescent not vaccinated.**	
Date of Collection	26 April 2020 to 5 June 2020
Number of specimens	85 (31 sera and 54 plasma)
Time after infection (gap elapsed between the date of the confirmatory RT-qPCR test and the sampling date)	
Range	0 to 139 days
Median	35.5 days
Number specimens with 0–30 days after infection.	26
Median	22 days
Number specimens with 31–60 days after infection	32
Median	37.5 days
Number specimens with >60 days after infection.	11
Median	84 days
Unknown	16
**Cohort-5: Pre-Pandemic Samples**	
**Subjects with unknown health status**	
Collection date	2012
Number included	78 (Sera)
**Other respiratory/viral infections**	
Collection date	2018 to 2019
Number included	47 (Sera)
Respiratory allergies	13
Zika virus	5
Dengue virus	5
Influenza A/B	12
Respiratory Syncytial Virus	6
Mycoplasma	6

**Table 2:** Characteristics of specimens from Cohorts-2 to 4 used in the study.

**Cohort-2: Serial samples from previously SARS-CoV-2 infected subjects that received two doses of mRNA vaccine (Pfizer-BioNTech or Moderna-1273).**	
Number of subjects	12
Sex	7 female and 5 males
Date of collection	27 October 2020 to 20 September 2021
Number of specimens	36
Sample 1 (Baseline)	Days after infection
Range	
Median	
Sample 2	Days after the 2nd dose
Range	15 to 32 days
Median	21.5
Sample 3	Days after the 2nd dose
Range	74 to 169 days
Median	96
**Cohort-3a: Serial samples from no previous SARS-CoV-2 infected subjects that received multiple Pfizer-BioNTech vaccinations.**	
Number of subjects	4
Sex	3 female and 1 male
Date of collection	10 August 2020 to10 August 2023
Number of specimens	26
Sample 1 (baseline)	Previous 1st dose
Sample 2	Days after the 2nd dose
Range	19 to 35 days
Median	20
Sample 3	Days after the 3rd dose
Range	31 to 43 days
Median	31
Sample 4	Days after the 3rd dose
Range	180 to 420 days
Median	255
Sample 5	Days after bivalent vaccine
Median	30 days
Sample 6	Days after bivalent vaccine
Range	90–180 days
Median	180
**Cohort-3b: Single sample from no previous SARS-CoV-2 infection multiple Pfizer-BioNTech vaccinations, collected ~2 years after the last dose.**	
Number of subjects	8
Sex	4 female and 4 males
Number of specimens	8
Date of sample collection	3 August 2023 to 23 October 2023
Date of last vaccination (3^rd^ dose or bivalent)	30 September 2021 to 28 December 2021
Sample 1	Days after the 3rd dose
Range	351 to 723 days
Median	634
**Cohort-4: Serial samples from no-previous SARS-CoV-2 infected subjects with inflammatory bowel disease (IBD) that received three doses of mRNA vaccine (Pfizer).**	
Number of subjects	6
Sex	3 female and 3 males.
Date of collection	14 April 2021 to 22 July 2022
Number of specimens	24
Sample 1 (baseline)	Days after 2nd dose
Range	15 to 28 days
Median	17
Sample 2	Days after 3rd dose
Median	60
Sample 3	Days after 3rd dose
Median	180

**Table 3:** Cohen’s Kappa analysis between antibody class/subclasses and their neutralization activity.

Antibody Class/Subclass	C-PASS Neutralization Test			
**Total IgG**	**WT**	**Alpha**	**Delta**	**Omicron**
% Agreement	98.87	75.29	69.41	3.529
Kappa Value	0.661	0.056	0.049	0.00057
Interpretation	Substantial agreement	Slight agreement	Slight agreement	No agreement
**IgG1**				
% Agreement	76.47	72.94	57.64	24.70
Kappa Value	0.050	0.268	−0.038	−0.016
Interpretation	Slight agreement	Fair agreement	No agreement	No agreement
**IgG2**				
% Agreement	9.41	31.76	34.88	--
Kappa Value	0.002	0.032	−0.014	No computed
Interpretation	No agreement	Slight agreement	No agreement	--
**IgG3**				
% Agreement	30.58	48.23	50.58	69.23
Kappa Value	0.018	0.151	0.147	0.006
Interpretation	Slight agreement	Slight agreement	Slight agreement	No agreement
**IgG4**				
% Agreement	14.11	30.588	35.29	--
Kappa Value	0.0064	0.011	−0.026	No computed
Interpretation	No agreement	Slight agreement	No agreement	--
**IgM**				
% Agreement	88.23	77.64	72.94	16.47
Kappa Value	0.255	0.298	0.266	0.0078
Interpretation	Fair agreement	Fair agreement	Fair agreement	No agreement
**IgA**				
% Agreement	40.00	55.29	48.23	64.19
Kappa Value	0.028	0.205	0.050	−0.002
Interpretation	Slight agreement	Fair agreement	Slight agreement	No agreement

**Kappa interpretation values:** 0.01–0.2 slight agreement. 0.21–0.4 fair agreement. 0.41–0.6 moderate agreement. 0.61–0.8 substantial agreement. 0.81–1.0 almost perfect or perfect agreement.

## Data Availability

All data are available upon request.

## Supplementary Materials

The following supporting information can be downloaded at: https://www.mdpi.com/article/10.3390/v16020187/s1, Figure S1: Deming analysis for serum/plasma equivalence and reproducibility of the in-house IgG ELISA; Table S1: Mean absorbance values obtained by in-house IgG ELISA for detection of IgG, and IgG1, IgG2, IgG3, and IgG4 antibody isotypes in samples (serum/plasma) collected from 85 non-hospitalized COVID-19 convalescent subjects; Table S2: Neutralization activity measured as surrogate virus neutralization percentage (sVNT%) against the wild-type, Alpha (B.1.1.7), Delta (B.1.617.2), and Omicron (B.1.1.529) variants of concern was determined by C-Pass method in samples (serum/plasma) collected from 85 non-hospitalized COVID-19 convalescent subjects; Table S3: Levels of IgG antibody subclasses to SARS-CoV-2 measured by in-house ELISAs and neutralizing percentages (sVNT%) measured by C-Pass surrogate virus neutralization test in serial samples collected from 12 COVID-19 convalescent subjects that received two doses of the mRNA vaccine (Pfizer–BioNTech or Moderna-1273); Table S4: Levels of IgG antibody subclasses to SARS-CoV-2 measured by in-house ELISAs and neutralizing activity measured by C-Pass surrogate virus neutralization assay (sVNT%) in serial samples collected from 14 subjects without previous SARS-CoV-2 infection who received multiple mRNA vaccinations (Pfizer–BioNTech or Moderna-mRNA-1273).
